# Health Tract, No. 120

**Published:** 1865

**Authors:** 


### COAL-FIRES.

WASHINGTON, when contemplating leaving home early of a winter's morning, would have every thing minutely arranged over-night, so that he might kindle his own fire, and thus avoid waking up the servants before daylight and depriving them of that necessary rest which was their due. Not long ago, a charity student in the —— Theological Seminary, of this city, on being enjoined to retire early and rise betimes, in order to save his eyes and health from the injurious effects of artificial light and late hours, complained that it was impracticable, because the colored man did not make the fires until seven o'clock, and that he himself did not know how to make a coal-fire. It would be too much to expect that such a youth will ever be more than a drone in the pulpit, or any where else than under an overseer on a well-conducted plantation. The writer's habit is to rise before day in fire-time of year, so as to be ready for study at the earliest moment of sufficient daylight; and for two other reasons: to allow the servants to get all the sleep they need, and to avoid that unwholesome impatience and annoyance which are sure to follow the oversleeping or unskillfulness of hirelings, for to begin a day in irritation spoils the head for study and the heart for happiness for hours if not for the entire day. To aid the servants, then, to perform a daily duty easily and well, to prevent those early irritabilities which are so apt to discompose whole families, and thus antagonize those mental serenities so essential to domestic comfort, to bodily health, and mental composure, it will be advantageous both to body and mind for every reader to become an adept in kindling a coal-fire. First, know that hard coal will not "get on fire" until it is thoroughly heated through and through; second, a small piece of coal does not require as much to heat it up as a larger piece, hence, the less wood you have to kindle with, the more necessary is it that the pieces of coal which are touching the wood should be small. As wood is more expensive than coal, economy suggests the use of as little wood as practicable. The coal, then, for kindling should not only be as small as a pigeon's egg, called "chestnut-coal" by the dealers, but to economize the wood, the pieces should not be over four inches long, so that they can be laid compactly, then the heat will be more concentrated on a given point of coal, and thus the sooner heat it through and through to the degree requisite for actual ignition. If the wood is thus placed and is covered with one layer of "chestnut" coal, it will redden with great rapidity and certainty; as soon as this is the case, cover over the reddened coal with another layer or two, and in a minute or two put on the larger size. By putting a handful of shavings or paper in a grate compactly, then some splinters of dry wood, not larger than the little finger, and outside of that a layer of pieces an inch or more thick and three or four long, then apply a match to the paper, and while it is "catching," put on the small coal as above, there will not be a failure during the winter, nor a fit of passion, nor a growl in the whole household, at least for want of a good and timely fire. To lessen a coal-fire, press it from the top, so as to make the mass more compact, giving less room for air; to revive it, lay on small pieces tenderly, put on the blower, and when red, add larger pieces and riddle out from below. Heaping on more coal or letting out the ashes below, will certainly put out a low coal-fire.

